# A new diagnostic autism spectrum disorder (DASD) strategy using ensemble diagnosis methodology based on blood tests

**DOI:** 10.1007/s13755-023-00234-x

**Published:** 2023-08-14

**Authors:** Asmaa H. Rabie, Ahmed I. Saleh

**Affiliations:** ComputerEngineering and Systems Dept., Faculty of Engineering, Mansoura University, Mansoura, Egypt

**Keywords:** Autism, Ensemble classification, Artificial intelligence, Feature selection, Outlier rejection

## Abstract

Autism Spectrum Disorder (ASD) is a complex neurodevelopmental disease that impacts a child’s way of behavior and social communication. In early childhood, children with ASD typically exhibit symptoms such as difficulty in social interaction, limited interests, and repetitive behavior. Although there are symptoms of ASD disease, most people do not understand these symptoms and therefore do not have enough knowledge to determine whether or not a child has ASD. Thus, early detection of ASD children based on accurate diagnosis model based on Artificial Intelligence (AI) techniques is a critical process to reduce the spread of the disease and control it early. Through this paper, a new Diagnostic Autism Spectrum Disorder (DASD) strategy is presented to quickly and accurately detect ASD children. DASD contains two layers called Data Filter Layer (DFL) and Diagnostic Layer (DL). Feature selection and outlier rejection processes are performed in DFL to filter the ASD dataset from less important features and incorrect data before using the diagnostic or detection method in DL to accurately diagnose the patients. In DFL, Binary Gray Wolf Optimization (BGWO) technique is used to select the most significant set of features while Binary Genetic Algorithm (BGA) technique is used to eliminate invalid training data. Then, Ensemble Diagnosis Methodology (EDM) as a new diagnostic technique is used in DL to quickly and precisely diagnose ASD children. In this paper, the main contribution is EDM that consists of several diagnostic models including Enhanced K-Nearest Neighbors (EKNN) as one of them. EKNN represents a hybrid technique consisting of three methods called K-Nearest Neighbors (KNN), Naïve Bayes (NB), and Chimp Optimization Algorithm (COA). NB is used as a weighed method to convert data from feature space to weight space. Then, COA is used as a data generation method to reduce the size of training dataset. Finally, KNN is applied on the reduced data in weight space to quickly and accurately diagnose ASD children based on new training dataset with small size. ASD blood tests dataset is used to test the proposed DASD strategy against other recent strategies [1]. It is concluded that the DASD strategy is superior to other strategies based on many performance measures including accuracy, error, recall, precision, micro_average precision, macro_average precision, micro_average recall, macro_average recall, F1-measure, and implementation-time with values equal to 0.93, 0.07, 0.83, 0.82, 0.80, 0.83, 0.79, 0.81, 0.79, and 1.5 s respectively.

## Introduction

ASD affects children’s understanding, communication, and behavior because it is linked to brain development [1–4]. ASD may begin in the early childhood for patients and then its effects persist until the end of the patient’s life. Children with ASD may have many symptoms that generally appear in the first two years of the patient's life. These symptoms such as difficulty learning, difficulty communicating with others, difficulty interacting with others, and repetitive behavior. A child with autism typically has a lack of emotional face when talking with others, spends a lot of time putting things in order, and feels hesitant [5, 6]. Actually, most people do not have enough knowledge of the symptoms of ASD to be able to diagnose a child with ASD or not. Additionally, individuals, families and, society spend a high cost in order to reduce and overcome ASD disease [1–4]. Hence, it is an important to find an ASD diagnostic model that can early diagnose ASD children before the ASD patient’s condition deteriorates.

Great efforts are being made by researchers in order to provide a rapid and accurate diagnosis model that can early detect patients who suffer from ASD with high efficiency to determine effective treatments, reduce cost, and control ASD disease. Initially, researchers did not have enough knowledge about the etiology of ASD to detect ASD cases related to blood tests [1]. Thus, they have relied to use diagnostic models that can provide a diagnosis of ASD children based on behavioral criteria using screening tools. Although several researches are based on using behavioral criteria to diagnose ASD patients, it is noted that it is a difficult way for determining behavioral criteria in younger children [1]. Recently, researchers have come to have a deep understanding of the etiology of ASD. Thus, many blood-based biomarkers have been used to correctly detect ASD cases [1]. AI techniques represent the most popular methods recently used to quickly and correctly detect ASD cases [7, 8].

Nowadays, AI techniques are used in medical analysis systems because these techniques have the ability to accurately analyze data, automatically find predictive information from big data, as well as extract unknown data [9–19]. In medical systems, AI can detect the hidden patterns of medical data and can also provide diagnosis [20–28]. AI includes many methods that can perform many tasks to serve many medical purposes. AI applications in medical systems such as resource demand analysis, disease diagnosis, pre-processing of non-informative features and invalid data, analysis of treatment costs, and forecasting of future disease outcomes [29–36]. Pre-processing and classification methods are among the most widely used AI methods to provide a fast and more accurate diagnostic model that can correctly diagnose diseases such as ASD disease.

A pre-processing process is used to eliminate irrelevant features and noise data from medical datasets prior to training the diagnostic method to enable it to give an accurate diagnosis with minimal time penalty [9–11]. Thus, the main aim of pre-processing process is to prevent overfitting and enable a diagnostic model to perform its tasks well. Two main processes called feature selection and outlier rejection should be performed on the collected dataset before it is passed to the diagnostic model. Feature selection is used to delete any irrelevant features while outlier rejection is used to keep only the valid training data [37, 38]. Feature selection methods are categorized into filter and wrapper categories [37, 38]. Outlier rejection methods are categorized into cluster, statistical, and neighbor categories [37, 38]. To diagnose diseases such as ASD disease, classification methods based on AI such as Bayesian classifiers, Association Rules, and neural networks can be used [11, 12]. The recent ASD diagnostic strategies cannot provide an optimal diagnosis. Hence, it is an important process to introduce a new diagnostic strategy that can provide a faster and more precise diagnosis. Accurate ASD diagnosis reduces cost and disease control.

In this paper, a new Diagnostic Autism Spectrum Disorder (DASD) strategy has been provided to quickly and priestly diagnose ASD patients. The main layers of DASD are (i) DFL which aims to filter dataset from both less important features and outliers to avoid the overfitting problem and (ii) DL which attempts to quickly and correctly detect ASD children depended on the followed dataset from DFL. In fact, BGWO is used as a feature selection method and BGA is used as an outlier rejection method to filter the dataset from any irrelevant features and any invalid training data before learning the diagnostic model in DL. In DL, EDM representing a new diagnostic model is used as the main contribution of this paper. In fact, EDM follows the ensemble classification principle, thus, it contains many diagnostic models where EKNN is one of them. The proposed EKNN diagnostic model contains NB as a weighted method, COA as a data generation method, and KNN as a diagnostic model which attempts to provide fast and accurate diagnosis based on the reduced dataset in weight space. Experimental results showed that DASD strategy outperforms other recent strategies as it provides the best accuracy, error, micro and micro average for precision and recall respectively, F1-measure, and implementation-time values.

The main contributions of this paper can be summarized in the following points:

(i) DASD is introduced as a new strategy consisting of two main layers called DFL and DL to early detect ASD cases in an accurate manner.
(ii) A new classifier, called EKNN, has been introduced, which employs three different mechanisms, namely; NB for feature weighting, COA for data generation, and KNN for diagnosing. Hence, it gives not only fast but also accurate results based on the reduced dataset.
(iii) A new diagnostic model called EDM, which employs the ensemble classification is provided to accurately diagnosing monkeypox cases. EDM is based on using several classifiers where the proposed EKNN is one of them. Hence, the decisions of the employed classifiers are combined based on majority voting method.

Based on experimental results, DASD strategy outperforms other recent strategies where it can provide fast and accurate results. In brief, this paper has been structured as follows; previous research efforts about diagnostic strategies for ASD is introduced in Sect. The Pros and Cons of the Proposed DASD Strategy. Section The Diagnostic Autism Spectrum Disorder (DASD) Strategy provides the suggested diagnostic autism spectrum disorder strategy while the introduced ensemble diagnosis method is discussed in details in Sect. The Proposed Ensemble Diagnosis Methodology (EDM). Section Experimental Results depicts the experimental results, Sect. The Pros and Cons of the Proposed DASD Strategy presents the pros and cons of the proposed DASD strategy, and Sect. “Conclusions and Future Directions” introduces the conclusions and future directions.

## The previous research efforts

In this section, a review of many previous ASD diagnostic models will be introduced. According to Alsaade et al. [2], three deep learning models called Visual Geometry Group Network (VGG19), NASNETMobile, and Xception were applied to detect ASD cases based on face recognition using dataset included many face images. At first, features were extracted from face images, then, the VGG19, NASNETMobile, and Xception was used on the extracted features to diagnose ASD patients. Experimental results proven that Deep Learning based on Xception (DL_ Xception) model outperformed other models; VGG19 and NASNETMobile because it introduced the best accuracy value. Although the efficiency of DL_Xception model, the preprocessing phase which includes feature selection as well as outlier rejection techniques was not used before using DL_Xception as a diagnostic model to give it the ability to give the best results. Additionally, this model has not been tested on the blood tests dataset.

According to Shuvo et al. [5], Random Forest (RF) diagnostic model was applied to diagnose ASD children based on ASD screening dataset that includes behavioral features on ASD patients. Initially, dataset was encoded to convert the nominal values to numerical values and then the repeat rows were removed. Finally, many decision trees were build using RF method to provide ASD diagnosis. Experimental results illustrated that RF method provided accurate diagnosis compared to other diagnostic models. Although RF is a simple method that can provide accurate results based on behavioral dataset, it provides different results according to different time. Thus, its parameters must be set to fixed values.Please confirm the section headings are correctly identified.ok

According to Ali et al. [6], ASD patients were diagnosed based on using structural Magnetic-Resonance Imaging (sMRI) model based on dataset included many brain images. At first, the sMRI model extracted features from brain images and then selected the most effective features for ASD patients. Finally, Artificial Neural Network (ANN) was applied to detect ASD cases. Based on experimental results, sMRI provided the best accuracy compared to other diagnostic models but its results were not the optimal because it only depended on using feature selection method without using outlier rejection method. Although ANN in sMRI model achieved high efficiency with the brain images dataset, it has not been tested on other datasets such as the blood tests dataset to prove its availability to deal with different datasets.

According to Ari et al. [7], the proposed ASD diagnostic model called Deep Convolutional Neural Network (DCNN) method was implemented on Electroencephalogram (EEG) signals. At first, data augmentation was performed before starting to use DCNN. Data augmentation was performed by using Extreme Learning Machines-based Auto Encoders (ELM-AE). Then, DCNN was applied to detect ASD cases according to their EEG signals. As reported in experimental results, it is concluded that DCNN provided more accurate results than other competitive methods. Although DCNN provided the best results, it could not reach to the optimal because it implemented on the original dataset without using preprocessing stage that includes feature selection and outlier rejection operations.Please confirm the placement of supplementary information link.ok

According to Hewitson et al. [1], blood tests dataset was applied to diagnose ASD cases using Logistic Regression Model (LRM). The implementation of LRM depended on correlation based method, RF, and t-test as a three different AI methods. LRM was implemented based on 9 selected proteins from 23 proteins in blood tests dataset to detect cases to ASD case class or Typically Developing (TD) case class. In experimental results, it is noted that the performance of LRM was better than other models. Although the benefits of LRM, it could not give the best results because it did not depend on preprocessing phase to filter the data before learning the AI methods.

According to Alkahtani et al. [39], a Convolutional Neural Network (CNN) model was introduced to improve ASD diagnosis. In fact, CNN was implemented on the behavior of patient and also the developmental history. Experimental results ensured that CNN based on MobileNet-V2 model can accurately detect ASD cases as it can give the highest accuracy. Although CNN outperformed other models, it should be combined with other artificial intelligence methods to improve the performance of the diagnosis. Additionally, CNN should be tested on different datasets with a large size.

According to Zhu et al. [40], a Response To Name (RTN) based on multimodal machine learning system was provided to accurately classify ASD disease using 125 toddlers where 61 of them are ASD, 31 of them are Developmental Delay (DD), and 33 of them are TD. Multimodal machine learning system has a significant impact on RTN where it can provide accurate results. Although Multimodal machine learning system provided accurate results, its effectiveness should be tested on different datasets with a large size.Kindly check and confirm whether the corresponding author mail id is correctly identified.ok

According to Saleh et al. [41], blood tests dataset was used to detect ASD patients using ASD Discovery (ASDD) a new strategy that depends on feature selection, outlier rejection, and diagnostic methods. In fact, fisher score was used to accurately select informative features and then hybrid bio-inspired optimization technique as a new outlier rejection method that uses genetic algorithm and grey wolf optimization algorithm in a binary version before learning the ensemble diagnostic model that includes three main classifiers. These classifiers are Naïve Bayes, K-Nearest Neighbors, and deep learning. The implementation results ensured that the proposed ASDD outperformed other strategies based on confusion matrix measures. Although the effectiveness of ASDD compared to other strategies, it should be developed to provide more accurate results at the minimum time.Please check the layout of Table 4, and correct if necessary.ok

## The diagnostic autism spectrum disorder (DASD) strategy

**Fig. 1:**
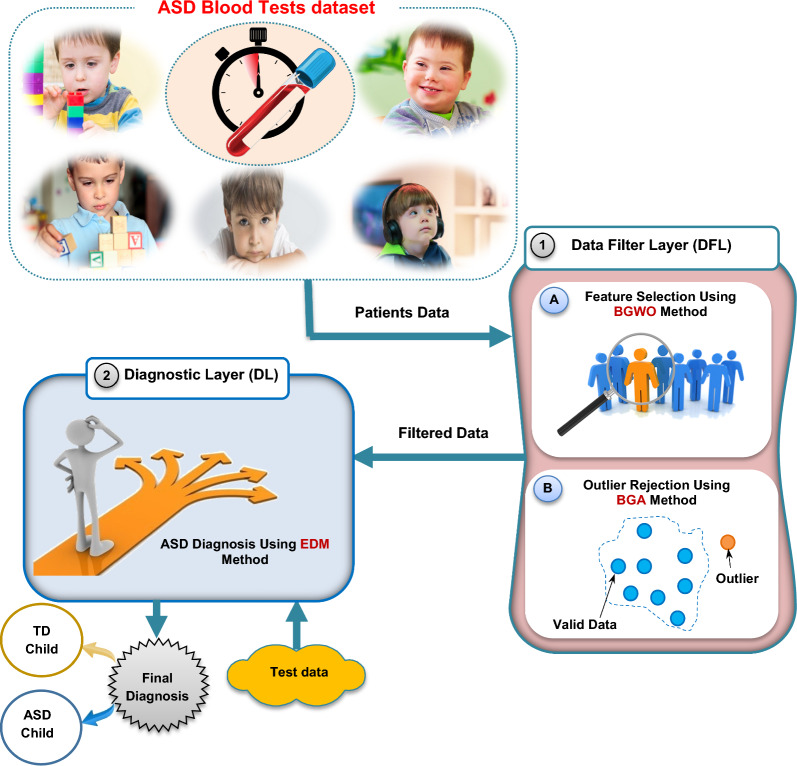
The diagnostic autism spectrum disorder (DASD) strategy

In this section, the proposed DASD strategy, as a new diagnostic strategy, will be discussed in detail. DASD is used to rapidly and precisely detect ASD cases based on an ASD dataset. In fact, the ASD dataset consists of blood tests from both ASD and TD cases. Figure 1 illustrates the main layers of DASD strategy, which are; Data Filter Layer (DFL) and Diagnostic Layer (DL). Filtering ASD dataset from non-informative features and invalid (outliers) data will be performed in DFL while the diagnostic model will be trained on the filtered data in DL to speedily and correctly diagnose ASD children. In DFL, feature selection and outlier rejection operations are used to filter the data before using the diagnostic model in the next layer called DL. In fact, irrelevant features will be eliminated using feature selection operation while valid data will be elected using outlier rejection operation. Then, the ASD dataset without irrelevant features and without outliers will be passed to DL to correctly learn the diagnostic model to give fast and accurate diagnosis.

According to feature selection process, the main categories of the selection methods are filter and wrapper [37, 38, 42]. Filter methods are fast but imprecise while wrapper methods are accurate but slower than filter methods [37, 38, 43, 44]. According to outlier rejection process, the main categories of the rejection methods are neighbor, cluster, and statistical methods [37, 38]. Nowadays, optimization techniques can be applied to choose the best features and also to reject invalid data. Although the execution time of optimization techniques may be large, they can provide more accurate results than other techniques [37, 38, 45]. In fact, more accurate results in DFL are more important than fast results because feature selection as well as outlier rejection methods are performed offline and also are performed before using the diagnostic technique. Hence, the implementation time of the diagnostic technique in DL is more important than the execution time of the processes of DFL. Accurate results of DFL allow the diagnostic model in DL to be trained on valid data and then can provide a rapid and accurate diagnosis. Accordingly, BGWO method is used as a wrapper selection method to select the best features that have an impact on ASD children [38]. On the other hand, BGA method is used as outlier rejection method to reject invalid data from the training dataset [37]. At the end, the filtered ASD dataset will be entered into DL to learn a new diagnostic model called EDM to introduce quick and more accurate diagnosis.

As shown in Fig. 1, there are many steps to perform the proposed DASD strategy to early detect ASD cases. At first, the collected dataset will be passed to the filter layer to remove irrelevant features by using BGWO and then the dataset with informative features will be passed to BGA to remove outliers. After that, the filtered dataset without non informative features and invalid data will be divided into training and testing data. The training data will be used to learn the EDM methodology and then the testing data will be used to test its performance by determining the final case diagnosis for a child with TD or ASD. The proposed EDM as a diagnostic method will be discussed in details through the next section.

## The proposed ensemble diagnosis methodology (EDM)

In this section, the proposed EDM, as a new diagnostic model, will be discussed in detail. In fact, EDM is implemented based on a valid dataset without outliers or irrelevant features after applying the filtering methods called BGWO and BGA in DFL. EDM is a diagnostic model applied to discover ASD cases related to the principle of ensemble classification. EDM consists of many classifiers where the proposed EKNN is one of these classifiers. In fact, EKNN is a hybrid diagnosis method that contains three essential techniques, which are; KNN [44, 45], NB as a weighted technique [44, 45], and COA as a data generation technique used to reduce the number of training data [46, 47]. The structure of EDM and the proposed EKNN are discussed in details in the next subsections.

### The structure of ensemble diagnosis methodology (EDM)

**Fig. 2:**
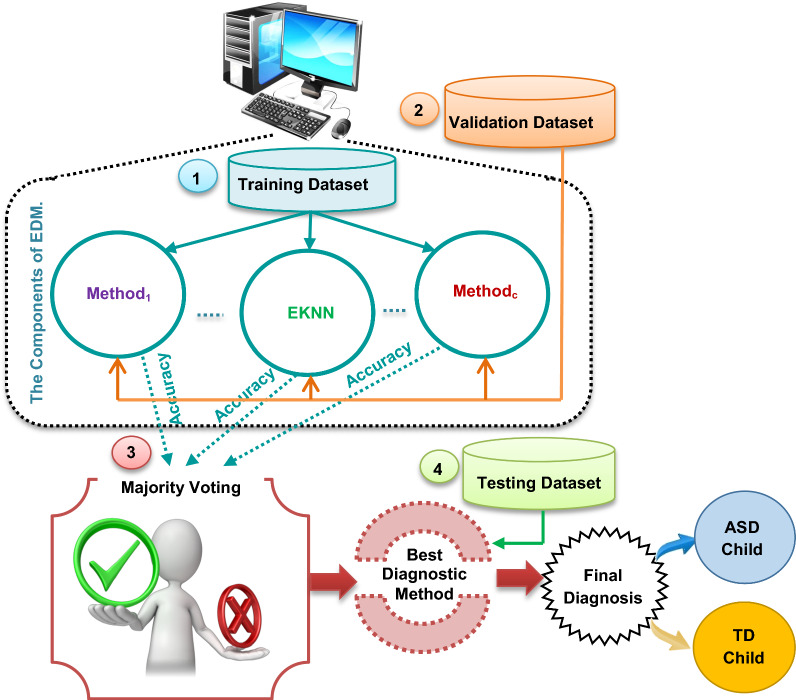
The structure of ensemble diagnosis methodology

In this subsection, the structure of the proposed EDM will be described. In fact, EDM consists of EKNN as a new classifier and many other classifiers as shown in Fig. 2. Figure 2 shows that the EDM method begins with training *‘c’* of classification (diagnostic) methods based on the ASD training dataset. In the second step, these methods are validated using the ASD validation dataset to calculate the accuracy of them based on confusion method [38]. In the third step, the accuracy values of these diagnostic methods are routed to a majority voting technique to determine the best well-trained method that can provide the best diagnosis. Finally, the ASD testing dataset is passed to the best diagnostic method to be diagnosed as ASD children or TD children.

After learning the ‘*c’* diagnostic methods, their accuracy values are represented as; *ACC* = *{ACC(Method*_*1*_*),…., ACC(EKNN),…., ACC(Method*_*c*_*)}*. These accuracy values are passed to a majority voting technique to find the well-trained method (*Best*_*method*_) based on the highest accuracy value using (1).

1 $${Best}_{method}=Method (High\left(ACC\right) )$$

where *Best*_*method*_ represents the best diagnostic method that achieve the highest accuracy value and *ACC* is a set of accuracy values for *‘c’* methods. *Method(High(ACC))* is the well-trained method that provides the highest accuracy value. At the end, the selected diagnostic method is used to give the best ASD diagnostic results based on testing data as illustrated in Fig. 2. In fact, in this paper, three main classifiers will be used in EDM, which are; Support Vector Machine (SVM) [48], Deep Learning Algorithm (DLA) [41], and the proposed EKNN. Through this work, DLA and SVM are used because these methods are the most recent used to diagnose ASD patients and have outperformed other diagnostic methods. To illustrate the idea of applying EDM, it is assumed that SVM, DLA, and EKNN provide accuracy values equal 75%, 82%, and 91% respectively. According to these accuracy values, the well-trained diagnostic method is EKNN because it can achieve the highest accuracy value. Hence, EKNN will be used to diagnose ASD patients.

Briefly, Fig. 2 illustrates the steps of learning the components of EDM methodology representing *‘c’* of diagnostic methods based on a part of dataset called training data. Then, these diagnostic methods are validated based on another part of dataset called validation data to determine the best diagnostic method provided in EDM that can accurately diagnose ASD cases. According to majority voting method, the diagnostic method that can give the maximum accuracy value will be used as the best method to provide the final diagnosis of ASD cases using the third and last part of the dataset called testing data. One of the diagnostic methods used in EDM is EKNN as a new method which will be discussed in detail in the next subsection.

### The enhanced K-nearest neighbors (EKNN) method

In this subsection, the proposed EKN, as a new method, will be explained in detail. This method includes KNN as a classifier [44, 45], NB as a weighted method [44, 45], and COA as a data generation technique used to reduce the number of training data [46, 47]. In fact, KNN is a simple and effective method, but it does not take in the account the effect or the weight of patient’s features on the class category. KNN is a lazy method, thus, it needs large execution time and high storage. Thus, KNN may be a slightly accurate method and needs to large time to be executed. In [44], KNN method has been integrated to NB classifier as a weighted method introducing a hybrid method called KN^3^B to take in the account the effect of features on the class category before applying KNN to provide more accurate results. Hence, KN^3^B can solve the first problem of KNN by providing more accurate results, but it cannot solve the second problem to reduce the execution time of KNN. Based on ASD dataset, the implementation of KN^3^B provided accurate diagnosis but it takes a long execution time.

**Fig. 3:**
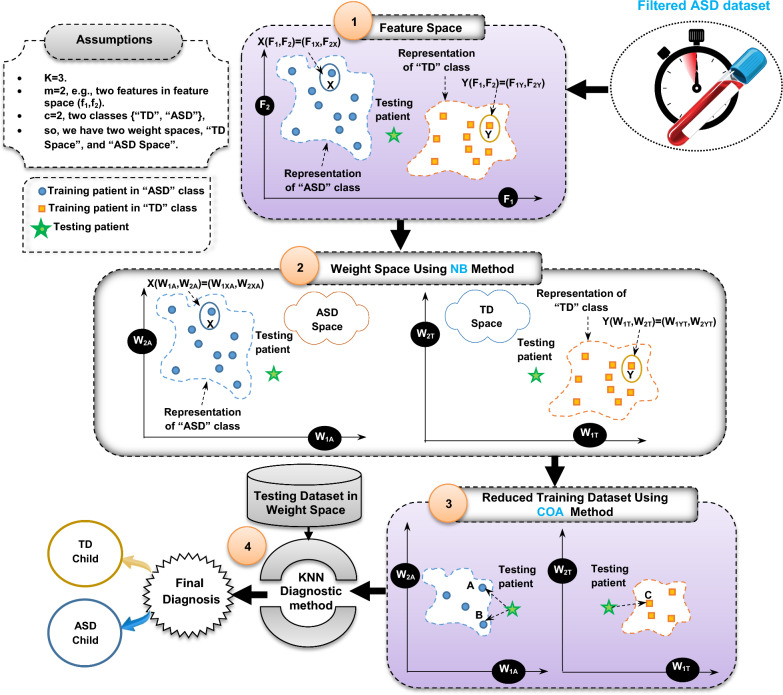
The sequential steps of implementing EKNN

**Fig. 4:**
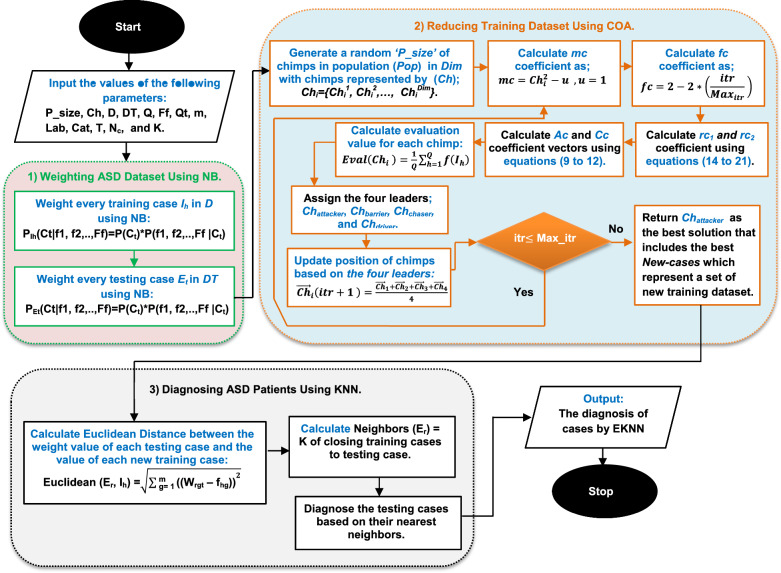
A flowchart of the EKNN content

Thus, KN^3^B method will be improved in this paper by adding COA as a data generation technique to it for reducing the number of training dataset before applying KNN method. The produced new method that includes these three techniques, which are; KNN, NB, COA is called EKNN that has the ability to provide a quick and accurate diagnosis. There are many sequential steps to implement the proposed EKNN as shown in Fig. 3. The pseudocode of EKNN is provided in algorithm 1. In fact, Fig. 3 and algorithm 1 describe four main stages to implement the proposed EKNN method. In the first stage, the filtered ASD dataset passed from DFL will be represented in the feature space according to two class categories, which are; “ASD” and “TD” assuming that there are two features in the feature space (F_1_, F_2_). In the second stage, NB as a weighted method will be implemented to convert the dataset from the feature space to the weight space at the second step. In other words, if each case belongs to ASD class is represented by X(F_1_, F_2_) = (F_1X_, F_2X_) and each case belongs to TD class is represented by Y(F_1_,F_2_) = (F_1Y_,F_2Y_) in the feature space, then these cases are represented in weight space as X(W_1A_, W_2A_) = (W_1XA_, W_2XA_) and Y(W_1T_, W_2T_) = (W_1YT_, W_2YT_) respectively. In the third stage, training dataset in the weight space will be reduced using COA. Finally, testing dataset will be diagnosed in weight space using KNN method based on the reduced training data. To clear the idea, Fig. 4 consists of a flowchart that describes the proposed EKNN method.

**Algorithm 1:**
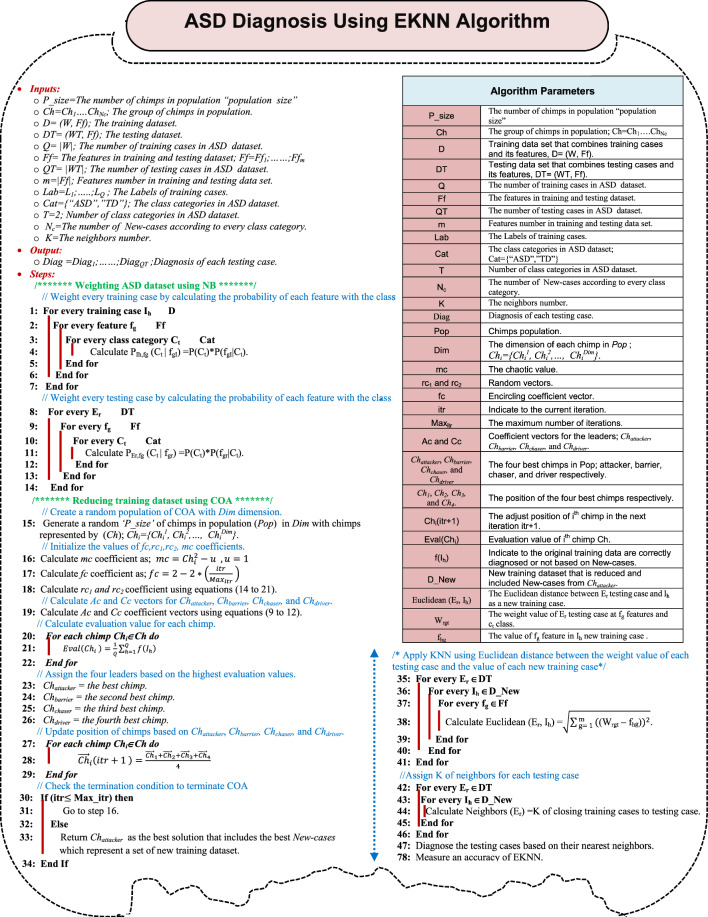
ASD diagnosis using EKNN algorithm

The EKNN is described in Fig. 4 through three stages, which are; weighting ASD dataset using NB, reducing training dataset using COA, and diagnosing ASD patients using KNN based on reduced training dataset that includes new training cases in weight space. COA is a metaheuristic algorithm that mimics the motivation behavior of chimps in groups for hunting the prey [46, 47]. COA is applied as a data generation technique in this paper to produce (not to select) the best set of training data for both class categories; ASD and TD. In the produced set of training data, the training cases are called “*New-cases*”. Thus, COA try to improve the KN^3^B by maximizing its accuracy value and minimizing the size of training data. A COA begins with Population (Pop) that includes a group of search agents (chimps) as potential solutions. The hunting process is performed by four groups of chimps called attacker, barrier, chaser, and driver which represent the fittest search agents in *Pop*. The first best chimp is called attacker (leader), the second one is barrier, the third one is chaser, and the fourth one is driver. According to the position of these four best chimps, the positions of the rest chimps in *Pop* will be updated.

**Fig. 5:**
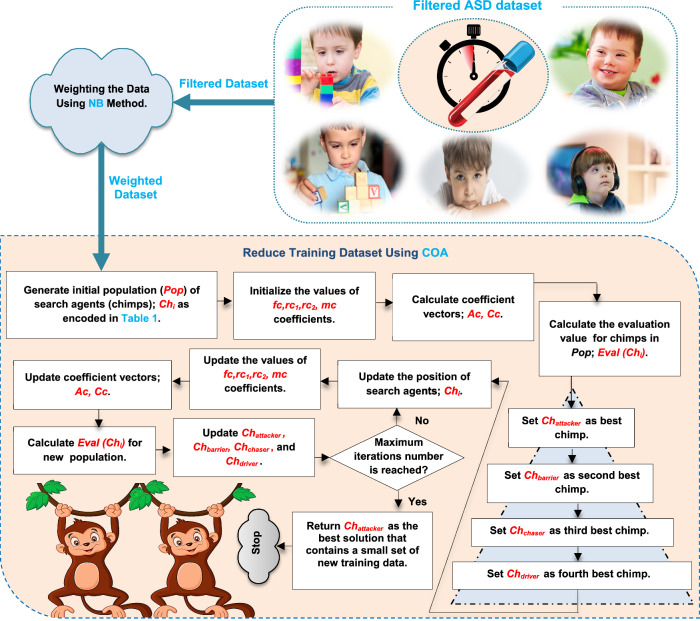
The sequential steps of implementing COA as a data generation method

The steps of implementing COA as data generation technique are presented in Fig. 5. According to Fig. 5, the first step is that search agents must be initialized in *Pop* where each agent is sequentially encoded as a set of *New-cases* for every class category. In fact, a set of *New-cases* in every agent is a complete solution to the reduction process. The encoding of each agent in *Pop* is showed in Table 1 where the dimension of each chimp is *Dim*; *Dim* = A*N_C_*Y_*D*_. Where *A* represents features number in each *New-case*, *N*_*C*_ represents *New-cases* number in each class, and* Y*_*D*_ represents class categories number that equal 2; *{ASD, TD}*. In Table 1, it is assumed that *A* = 4 and *N*_*C*_ = 2, hence, *Dim* = 4*2*2 = *16*. Accordingly, the dimension of each search agent (chimp) equal 16. The position (feature) value of *ith* chimp is represented as *Ch*_*fp*_*(jl)* which represents the value of *pth* feature in chimp according to *jth* New-case at *lth* class where p = {1,2,…,4}, j = {1,2}, and l = {1,2} = {“ASD”, “TD”}. Additionally, *New-case*_*jl*_ refers to the *jth* New_case at *lth* class.

**Table1:** The encoding of a single chimp in COA

	New-cases (ASD)								New-cases (TD)							
	New-case11				New-case21				New-case12				New-case22			
Chimp i	F1	F2	F3	F4	F1	F2	F3	F4	F1	F2	F3	F4	F1	F2	F3	F4
	Chf1(11)	Chf2(11)	Chf3(11)	Chf4(11)	Chf1(21)	Chf2(21)	Chf3(21)	Chf4(21)	Chf1(12)	Chf2(12)	Chf3(12)	Chf4(12)	Chf1(22)	Chf2(22)	Chf3(22)	Chf4(22)

According to the second step in Fig. 5, search agents are evaluated after initialization in *Pop* using evaluation function that measures the effectiveness of KN^3^B (weighted KNN) based on *New-cases* of *ith* search agent (Ch_i_). Calculating of the evaluation value for each search agent (*Eval(Ch*_*i*_*)*) can be performed using (2).

2 $$Eval\left({Ch}_{i} \right)=\frac{1}{Q}\sum_{h=1}^{Q}f\left({I}_{h}\right)$$

where the training cases number in ASD dataset is *Q* and *h* is an index that indicates to each training case in the ASD dataset.* f(I*_*h*_*)* refers to the correctly diagnosis of the training case* I*_*h*_ based on the *New-cases* in the *Ch*_*i*_ agent using KN^3^B. The corresponding class category of each training case* I*_*h*_ is determined based on the nearest *K* of the *New-cases* encoded in *Ch*_*i*_. In fact, *f(I*_*h*_*)* equal 1 in the case if *I*_*h*_ is correctly diagnosed, otherwise, *f(I*_*h*_*)* equal 0. COA searches for the optimal search agent (optimal *New-cases*) with the aim of increasing *f(I*_*h*_*)*. Based on the evaluation values of the search agents in *Pop*, the four best solutions (leaders) are determined; *Ch*_*attacker*_,* Ch*_*barrier*_*, Ch*_*chaser*_*,* and* Ch*_*driver*_. Based on the positions of these four leaders at each iteration (*itr*), the rest of search agents (*Ch*_*i*_) in *Pop* will be updated for the next iteration (*itr* + *1*) using (3–7) [46, 47].

3 $$\begin{array}{*{20}c} {\overrightarrow {Ch}_{1} \left( {itr + 1} \right) = \overrightarrow {Ch}_{attcker} \left( {itr} \right) - \overrightarrow {Ac}_{1} \overrightarrow {.Dc}_{attcker} ,\,} & {\overrightarrow {Dc}_{attcker} = \left| {\overrightarrow {Cc}_{1} .\overrightarrow {Ch}_{attcker} - mc*\overrightarrow {Ch}_{i} \left( {itr} \right)} \right| } \\ \end{array}$$

4 $$\begin{array}{*{20}c} {\overrightarrow {Ch}_{2} \left( {itr + 1} \right) = \overrightarrow {Ch}_{barrier} \left( {itr} \right) - \overrightarrow {Ac}_{2} \overrightarrow {.Dc}_{barrier} ,\,} & {\overrightarrow {Dc}_{barrier} = \left| {\overrightarrow {Cc}_{2} .\overrightarrow {Ch}_{barrier} - mc*\overrightarrow {Ch}_{i} \left( {itr} \right)} \right|} \\ \end{array}$$

5 $$\begin{array}{*{20}c} {\overrightarrow {Ch}_{3} \left( {itr + 1} \right) = \overrightarrow {Ch}_{chaser} \left( {itr} \right) - \overrightarrow {Ac}_{3} \overrightarrow {.Dc}_{chaser} ,\,} & {\overrightarrow {Dc}_{chaser} = \left| {\overrightarrow {Cc}_{3} .\overrightarrow {Ch}_{chaser} - mc*\overrightarrow {Ch}_{i} \left( {itr} \right)} \right|} \\ \end{array}$$

6 $$\begin{array}{*{20}c} {\overrightarrow {Ch}_{4} \left( {itr + 1} \right) = \overrightarrow {Ch}_{driver} \left( {itr} \right) - \overrightarrow {Ac}_{4} \overrightarrow {.Dc}_{driver} ,\,} & {\overrightarrow {Dc}_{driver} = \left| {\overrightarrow {Cc}_{4} .\overrightarrow {Ch}_{driver} - mc*\overrightarrow {Ch}_{i} \left( {itr} \right)} \right| } \\ \end{array}$$

7 $${\overrightarrow{Ch}}_{i}\left(itr+1 \right)=\frac{{\overrightarrow{Ch}}_{1}+{\overrightarrow{Ch}}_{2}+{\overrightarrow{Ch}}_{3}+{\overrightarrow{Ch}}_{4}}{4}$$

where *itr* indicates to the number of current iteration, *Ch*_*i*_* (itr)* is the position of each agent in iteration *itr*, and *DC* represents the distance between the search agent (*Ch*_*i*_) and a pray. *Ch*_*1*_*, Ch*_*2*_*, Ch*_*3*_*,*and* Ch*_*4*_ are the positions of the four leaders respectively. *mc* is a chaotic value between 0 and 1 using quadratic map that indicates to the effect of the chimps’ sexual motivation calculated using (8).

8 $$mc={Ch}_{i}^{2}-u , u=1$$

Additionally, *Ac* and *Cc* are coefficient vectors updated to find a solution close to the best solution that can be calculated for each leader using (9–12).

9 $$\begin{array}{*{20}c} {Ac_{1} = \left| {2*fc*rc_{11} - fc} \right|,\,} & {Cc_{1} = 2*rc_{12} } \\ \end{array}$$

10 $$\begin{array}{*{20}c} {Ac_{2} = \left| {2*fc*rc_{21} - fc} \right|,\,} & {Cc_{2} = 2*rc_{22} } \\ \end{array}$$

11 $$\begin{array}{*{20}c} {Ac_{3} = \left| {2*fc*rc_{31} - fc} \right|,} & {Cc_{3} = 2*rc_{32} } \\ \end{array}$$

12 $$\begin{array}{*{20}c} {Ac_{4} = \left| {2*fc*rc_{41} - fc} \right|,\,} & {Cc_{4} = 2*rc_{42} } \\ \end{array}$$

where *fc* is linearly decreasing from 2 to 0 and it is calculated using (13).

13 $$fc=2-2*(\frac{itr}{Max\_itr} )$$

where *Max_itr* is the maximum iterations number. *rc*_*1*_ and *rc*_*2*_ represent random factors between 0 and 1 which can be measured for each leader using (14–21) [47].

14 $$\begin{array}{*{20}c} {rc_{11} = k_{1} g_{1} *Random(),\,} & {k_{1} g_{1} = 1.95 - \left( {\frac{{2*\left( {itr^{\frac{1}{4}} } \right)}}{{Max\_itr^{\frac{1}{3}} }} } \right)} \\ \end{array}$$

15 $$\begin{array}{*{20}c} {rc_{12} = k_{2} g_{1} *Random(),\,} & {k_{2} g_{1} = \left( {\frac{{2*\left( {itr^{\frac{1}{3}} } \right)}}{{Max\_itr^{\frac{1}{3}} }} } \right) + 0.5} \\ \end{array}$$

16 $$\begin{array}{*{20}c} {rc_{21} = k_{1} g_{2} *Random(),\,} & {k_{1} g_{2} = 1.95 - \left( {\frac{{2*\left( {itr^{1/3} } \right)}}{{Max_{ - } itr^{1/4} }}} \right)} \\ \end{array}$$

17 $$\begin{array}{*{20}c} {rc_{22} = k_{2} g_{2} *Random(),\,} & {k_{2} g_{2} = \left( {\frac{{2*\left( {itr^{3} } \right)}}{{Max\_itr^{3} }} } \right) + 0.5} \\ \end{array}$$

18 $$\begin{array}{*{20}c} {rc_{31} = k_{1} g_{3} *Random(),\,} & {k_{1} g_{3} = \left( {\frac{{ - 3*\left( {itr^{3} } \right)}}{{Max\_itr^{3} }} } \right) + 1.5} \\ \end{array}$$

19 $$\begin{array}{*{20}c} {rc_{32} = k_{2} g_{3} *Random()} & {k_{2} g_{3} = \left( {\frac{{2*\left( {itr^{\frac{1}{3}} } \right)}}{{Max\_itr^{\frac{1}{3}} }} } \right) + 0.5} \\ \end{array}$$

20 $$\begin{array}{*{20}c} {rc_{41} = k_{1} g_{4} *Random()} & {k_{1} g_{4} = \left( {\frac{{ - 2*\left( {itr^{3} } \right)}}{{Max\_itr^{3} }} } \right) + 0.5} \\ \end{array}$$

21 $$\begin{array}{*{20}c} {rc_{42} = k_{2} g_{4} *Random(),\,} & {k_{2} g_{4} = \left( {\frac{{2*\left( {itr^{3} } \right)}}{{Max\_itr^{3} }} } \right) + 0.5} \\ \end{array}$$

where *Random()* is uniform distribution between 0 and 1. Additionally, *k*_*1*_*g*_*1*_*, k*_*2*_*g*_*1*_*, k*_*1*_*g*_*2*_*, k*_*2*_*g*_*2*_*, k*_*1*_*g*_*3*_*, k*_*2*_*g*_*3*_*, k*_*1*_*g*_*4*_*,* and *k*_*2*_*g*_*4*_ are dynamic coefficients used to calculate *rc*_*1*_ and *rc*_*2*_. In fact, to adjust the positions of search agents in *Pop*, a probability of 50% is assumed to choose between either the chaotic model (*mc*) or the normal adjusting position method using (22).

22 $${Ch}_{i}(itr+1)=\left\{\begin{array}{c}\frac{{Ch}_{1}+{Ch}_{2}+{Ch}_{3}+{Ch}_{4}}{4}, if( z<0.5)\\ \\ mc , if( z\ge 0.5)\end{array}\right.$$

where *z* is a random value between 0 and 1. These steps will be continued until the stopping criteria or the maximum iterations number is satisfied. Hence, in brief, COA begins by generating a random population and putting the positions of the leaders (*Ch*_*1*_*, Ch*_*2*_*, Ch*_*3*_*,*and *Ch*_*4*_) to zero vector. Secondly, each search agent (solution) in *Pop* is evaluated using (2) and then the best four search agents which provide the highest evaluation values will be assigned as leaders. Thirdly, the positions of the leaders (*Ch*_*1*_*, Ch*_*2*_*, Ch*_*3*_*,*and *Ch*_*4*_) will be updated using (3–6). Fourthly, the value of* mc* is adjusted using (8) and also the values of *fc, rc*_*1*_*,* and *rc*_*2*_ are adjusted using (13–21). Based on the values of *fc, rc*_*1*_*, rc*_*2*_*,* and* mc*, the values of *Ac* and *Cc* are adjusted using (9–12). At the end, the positions of search agents are adjusted using (3–7) and (21). If the stopping conditions does not satisfied, these steps will be repeated. Otherwise, the attacker (leader) will be introduced as the best solution. The *New-cases* in attacker chimp will be used as a new training dataset that have a smaller size than the original training dataset equal to v; v < Q, where Q is the original size of the training dataset.

Now, it is time to use KNN as a diagnostic method based on the new training dataset that is generated after weighting and minimizing the original training data. From the foregoing, it was concluded that the diagnosis process using KNN does not performed until the process of weighting data and minimizing training dataset are performed. Thus, the execution time of KNN based on the weighted and minimized dataset is more important than the execution time of COA to reduce the training dataset because it is an offline stage before using KNN to diagnose the patients. Thus, the execution time of COA does not affect KNN execution time but COA provides a small set of training dataset to minimize the execution time of KNN and enable it to provide rapid and precise diagnosis. In the next section, the proposed EKNN will be tested against other diagnostic methods and then the proposed DASD will be tested against other strategies.

## Experimental results

In this section, the proposed DASD strategy will be implemented and tested against other strategies for early diagnose of ASD patients. There are many followed steps to implement the DASD strategy that begins with executing BGWO to identify the most useful features and then executing BGA to remove outliers from the ASD dataset in DFL. At the end, the valid dataset without irrelevant features or outliers are passed to DL to correctly learn a new diagnostic technique called EDM. In this implementation, EDM consists of three main diagnostic models, which are; SVM [48], DLA [41], and the proposed EKNN. In fact, the implementation of DASD strategy will be performed through two main scenarios. Initially, the three methods of EDM, which are; SVM, DLA, and EKNN will be implemented in the first scenario and compared with other methods called NB and traditional KNN [44, 45]. In the second scenario, the DASD strategy based on the best provided diagnostic model from the first scenario will be tested and compared with several recent diagnostic strategies. In this implementation, the ASD dataset consisting of blood tests from TD and ASD cases is used where TD refers to healthy children without autism disease but ASD refers to children with autism disease [1, 41]. Accuracy, error, recall, precision, micro and macro average for precision and recall respectively, and F1-measure are used as performance measures based on the confusion matrix to calculate the performance of the applied techniques [9–11]. Additionally, tenfold cross-validation method is used to divide the dataset into ten equal groups where nine of them are applied as training sets and the other set is applied as a testing set. The values assigned to the used parameters are listed in Table 2.

**Table 2:** The assigned values for the used parameters

Parameter	Description	Applied value
Pcrossover	Probability of crossover in BGA	Random (0 ≤ Pcrossover ≤ 1)
Pmutation	Probability of mutation in BGA	Random (0 ≤ Pmutation ≤ 1)
Pselection	Probability of selection in BGA	Random (0 ≤ Pselection ≤ 1)
rand1 and rand2	Random numbers in BGWO	Random (0 ≤ rand1,rand2 ≤ 1)
a	Linearly decrease in BGWO	[2,0]
Max_iter	The maximum iterations number in BGWO,BGA, and COA	100
Random ()	Uniform distribution value in COA	Random (0 ≤ Random () ≤ 1)
z	The random value to choose between the chaotic model or the normal adjusting position method	Random (0 ≤ z ≤ 1)
K	The closed number of neighbors	1 ≤ K ≤ 5
C	The cost parameter used in linear kernel for SVM	[2–5, 215]
R	The learning rate value for DLA	0.01
U1, U2, and U3	The number of LSTM units in each layer of DLA	[32, 64, 128]
H	The number of hidden layers of DLA	1
D1, D2, and D3	The dropout rate in each layer of DLA	[0.2, 0.4, 0.6]

**Fig. 6:**
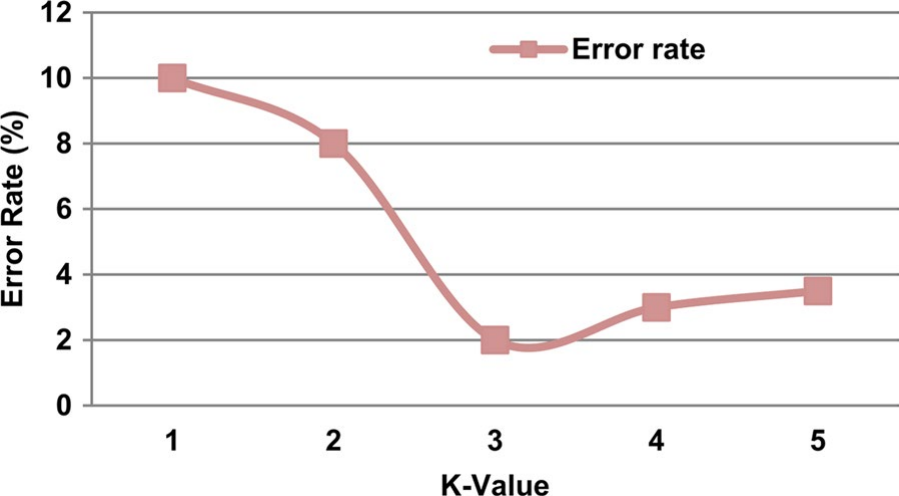
Error rate at each value of K

As provided in Table 2, the values for the parameters of BGWO, BGA, COA are randomly assigned according to each iteration. These parameters are *P*_*crossover*_*, P*_*mutation*_*, P*_*selection*_*, rand*_*1*_*, rand*_*2*_*, a, Random (),* and* Z*. In the most optimization algorithm researches, the best maximum iterations number is 100, thus, *Max_iter* for all optimization algorithms (BGWO, BGA, and COA) equals 100. According to the value of K, it is determined experimentally in the range [1–5] based on the use of 100 cases from the ASD dataset where 70 of them are training cases and 30 are testing. In fact, the accuracy of KNN and its error value are calculated based on each value of K where the best value of K is the value that maximizes the KNN’s accuracy and minimizes its error value. In this work, the best value of K is 3 as it can provide the minimum error value as illustrated in Fig. 6. Consequently, K = 3 will be used in the next experiments. In this work, the best value of C for SVM is 16. To implement DLA, the learning rate value is 0.01 (R = 0.01), the best number of LSTM units in each layer of DLA input (U_1_), hidden (U_2_), and output (U_3_) are 32, 64, and 128 respectively, and the best number of hidden layers is one layer. Additionally, the dropout rate in each layer of DLA input (D_1_), hidden (D_2_), and output (D_3_) are 0.2, 0.4, and 0.6 respectively.

**Fig. 7:**
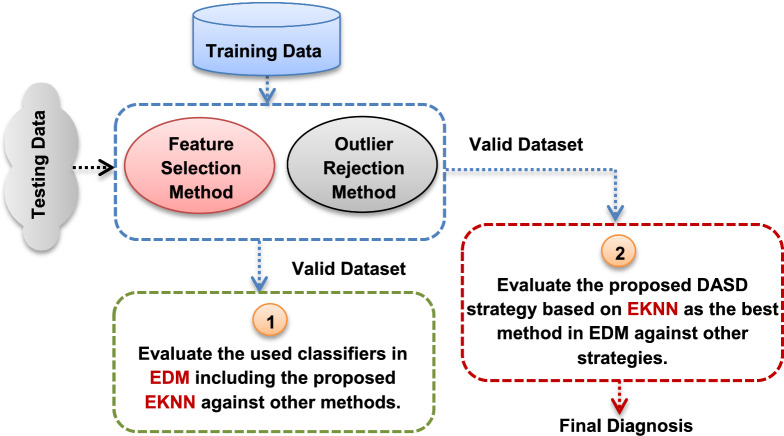
The two main steps of evaluating the proposed DASD strategy

Actually, the evaluation of the proposed work is performed through two main steps as shown in Fig. 7. In the first step, the proposed EKNN will be tested against the standard NB, KNN, and the other classifiers used in the EDM, namely SVM and DLA based on a valid dataset without outliers or irrelevant features to ensure the performance of EKNN against other diagnostic methods. Then, in the second step, the performance of the proposed DASD strategy based on EKNN will be tested against other diagnostic strategies. In fact, the simulation was done on one platform using MATLAB 2018a installed on a laptop depending on Intel (R) Core (TM) i5-10210U and @2.11G with 16.0 GB of RAM. Additionally, this laptop have Windows 10 (64 bit) operating system.

### The description of ASD dataset

**Fig. 8:**
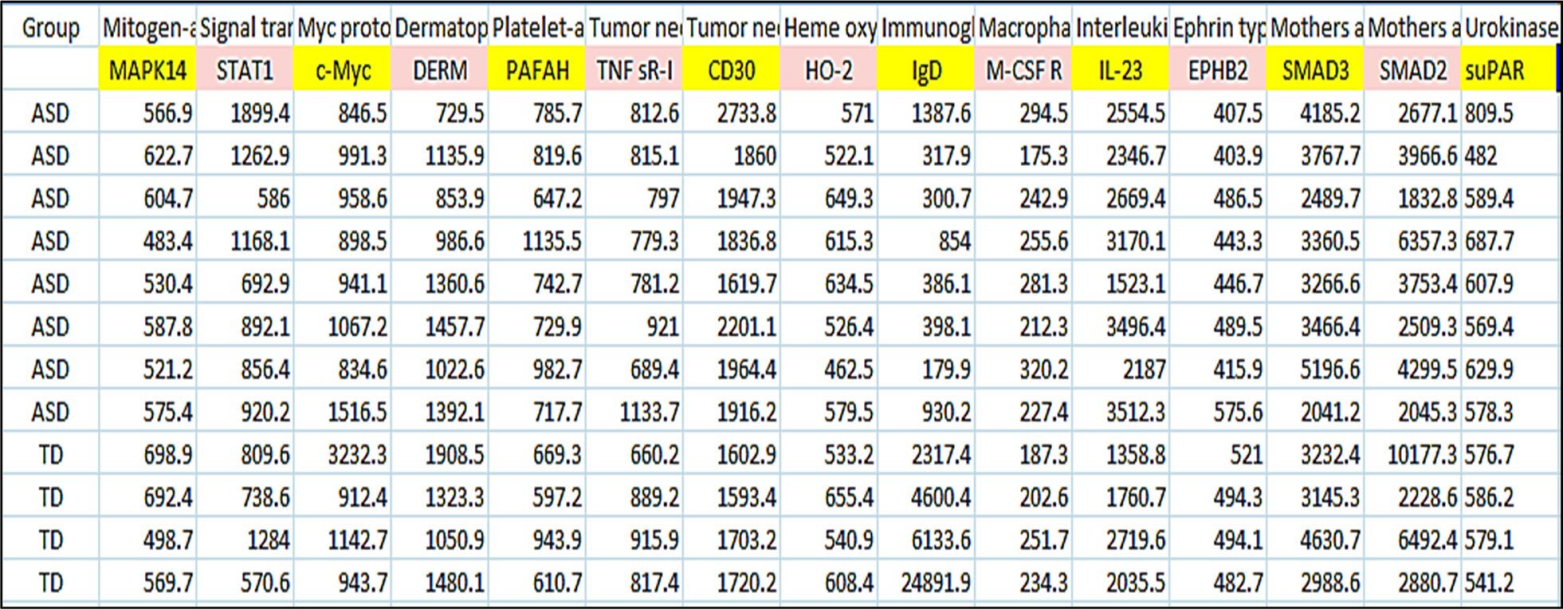
A snapshot from the dataset

In this subsection, the ASD dataset, which includes a set of blood test data collected from both autistic children (ASD) and non- autistic children (TD) in the age range of 18 months to 8 years, will be described in detail [1, 41]. This dataset contains the analysis of the levels of many proteins in plasma/serum changed in ASD patients. This dataset contains a total number of patients equal 154 cases according to the analysis of 1125 features (proteins) where 76 cases are ASD children and 78 cases are TD children. These proteins have been classified after careful examination into psychiatric medications, age, co-morbid conditions, and ethnicity classes [1]. Psychiatric medications class consists of 7 proteins called *{None, Anti-psychotic, Anti-depressant, SSRI, Stimulant, Sedative, Not reported}*. Age class consists of 1 protein that indicates to the ages of patients. Additionally, co-morbid conditions class consists of 9 proteins called *{None, Asthma, Sleep Apnea, Seasonal Allergies, GERD, Celiac Disease, PTSD, ADHD, Not reported}*. Ethnicity class consists of 6 proteins called *{Hispanic/Latino, American/Black, Multiple ethnicities or Other, White/Caucasian, African Asian or Pacific Islander, Not reported}*. Figure 8 shows a snapshot from the dataset. In this snapshot (Fig. 8), the columns consists of the selected proteins (features) that have an effect on ASD cases according to their blood tests while the rows consists of TD and ASD cases diagnosed based on their measurements based on their features.

The description of the selected features using BGWO, which equal 15 features, are provided in Table 3. In Table 3, the name, symbol, and description of each feature is represented in the rows of this table. The distribution of cases in ASD dataset based on the disease is provided in Table 4. As presented in Table 4, the number of ASD cases is 76 while the number of TD cases is 78. Additionally, the mean age for ASD is 5.6 years while the mean age for TD is 5.7. According to the ethnic breakdown, the number of African American/Black is 17, White/Caucasian is 73, Asian or Pacific Islander is 5, Multiple ethnicities or other is 23, Hispanic/Latino is 32, and not reported is 4.

**Table 3:** The selected features in ASD dataset by BGWO and their description

Feature	Symbol	Description
Mitogen-activated protein kinase 14	MAPK14	It is an enzyme represented in humans by a gene called MAPK14 known as p38-α
Signal transducer and activator of transcription 1-alpha/beta	STAT1	It is a transcription factor represented in humans by a gene called STAT1
Myc proto-oncogene protein	c-Myc	It represents a multifunctional, nuclear phosphoprotein that controls a many different cellular functions
Dermatopontin	DERM	It is a protein represented in people by a gene called DPT
Platelet-activating factor acetylhydrolase	PAFAH	It is a platelet aggregation and granulolytic agent, a potent phospholipid activator and a mediator of several leukocyte functions, inflammation, and anaphylaxis
Tumor necrosis factor receptor superfamily member 1A	TNF sR-I	It is a member of the tumor necrosis factor receptor superfamily of proteins
Tumor necrosis factor receptor superfamily member 8	CD30	It is a cell membrane protein of the tumor necrosis factor receptor family
Heme oxygenase 2	HO-2	It is an enzyme represented in people by a gene called HMOX2
Immunoglobulin D	IgD	It is an antibody that is 0.25% of the immunoglobulins present in the blood serum
Macrophage colony-stimulating factor 1 receptor	M-CSF R	It is a cell-surface protein represented in people by a gene called CSF1R
Interleukin-23	IL-23	It is a heterodimeric cytokine containing an IL-12B (IL-12p40) subunit and an IL-23A (IL-23p19) subunit
Ephrin type-B receptor 2	EPHB2	It is a protein represented in people by a gene called EPHB2
Mothers against decapentaplegic homolog 3	SMAD3	It is a member of the SMAD family of proteins initiated by the transforming growth factor beta (TGF-β)
Mothers against decapentaplegic homolog 2	SMAD2	It is a protein from the SMAD family determined in Drosophila
Urokinase plasminogen activator surface receptor	suPAR	It is a protein represented in people by a gene called PLAUR

**Table 4:** Distribution of cases in ASD dataset based on the disease

Criteria	Value/Description			
Total number of cases	ASD		TD	
	76		78	
Mean age	ASD		TD	
	5.6 years		5.7 years	
Ethnic breakdown	White/Caucasian	Hispanic/Latino		African American/Black
	73	32		17
	Asian or Pacific Islander	Multiple ethnicities or other		Not reported
	5	23		4

### Testing the ensemble diagnosis methodology (EDM)

**Fig. 9:**
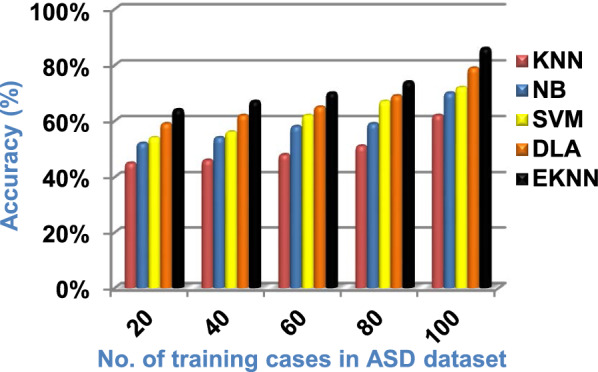
Accuracy of the used diagnostic methods

**Fig. 10:**
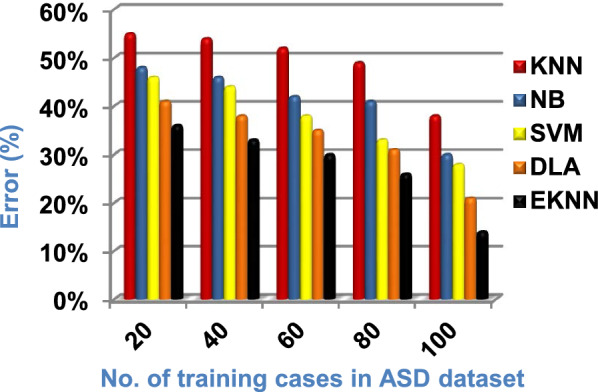
Error of the used diagnostic methods

**Fig. 11:**
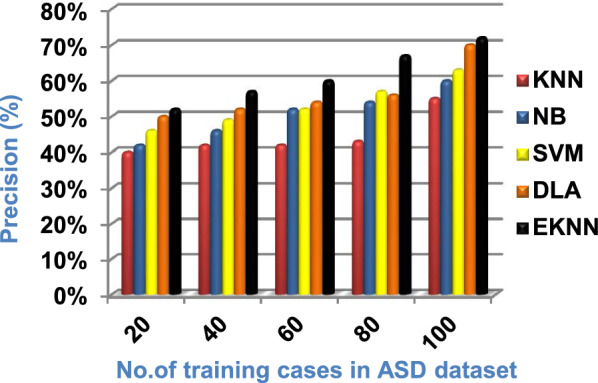
Precision of the used diagnostic methods

**Fig. 12:**
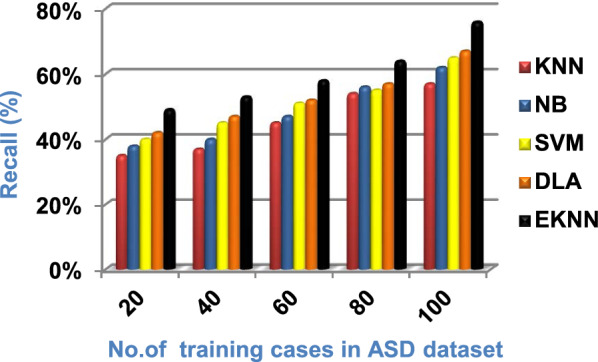
Recall of the used diagnostic methods

**Fig. 13:**
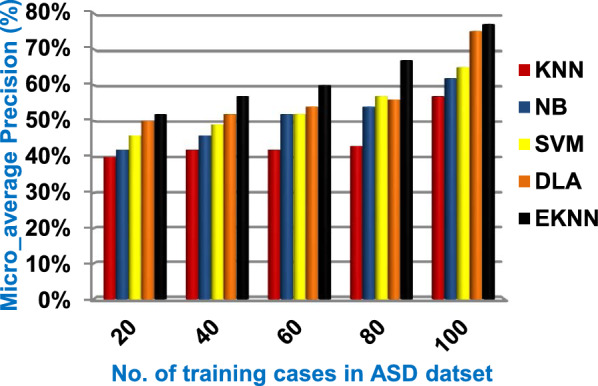
Micro_average precision of the used diagnostic methods

**Fig. 14:**
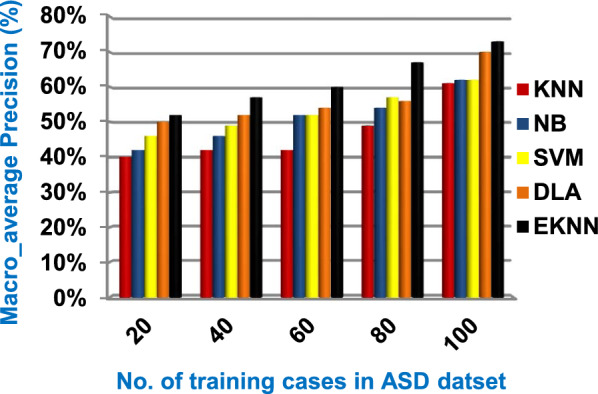
Macro_average precision of the used diagnostic methods

**Fig. 15:**
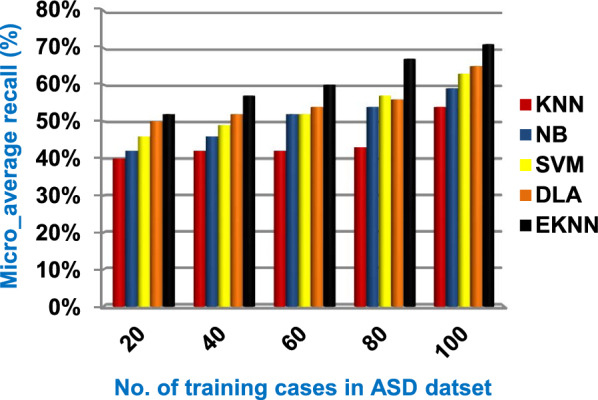
Micro_average recall of the used diagnostic methods

**Fig. 16:**
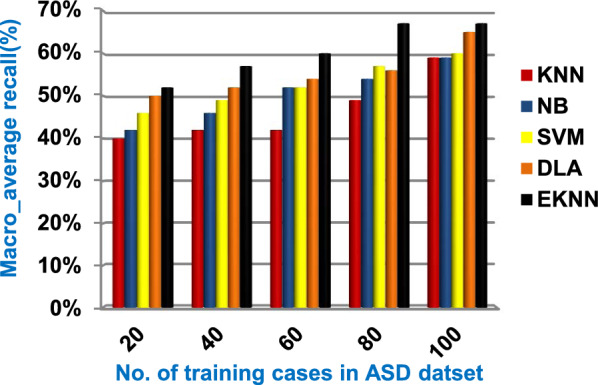
Macro_average recall of the used diagnostic methods

**Fig. 17:**
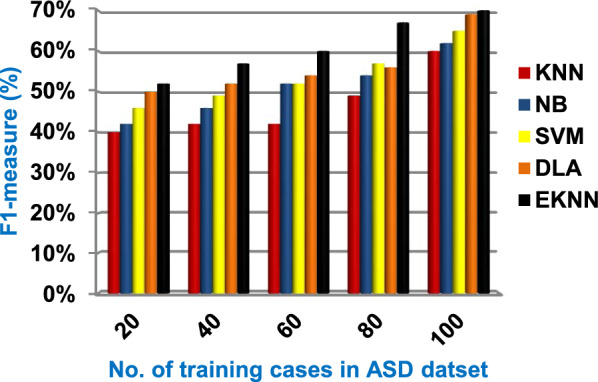
F1-measure of the used diagnostic methods

**Fig. 18:**
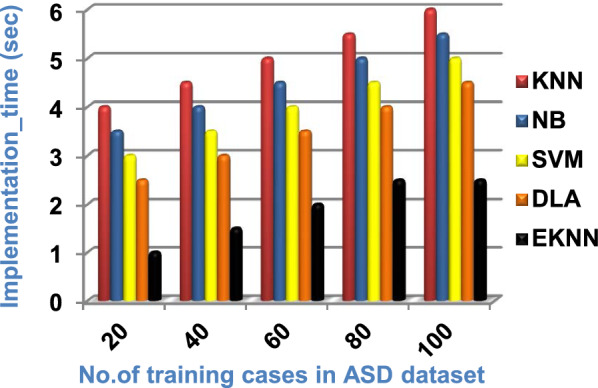
Implementation time of the used diagnostic methods

In this section, a new diagnostic model called EDM will be tested against several modern diagnostic models to determine the best model that can provide accurate diagnosis. Hence, the three methods of EDM, which are; SVM, DLA, and the proposed EKNN are compared to NB and the classical KNN. The accuracy, error, recall, precision, micro and macro average for precision and recall respectively, F1-measure, and implementation-time of these diagnostic methods are showed in Figs. (9, 10, 11, 12, 13, 14, 15, 16, 17, 18) and Table 5. In fact, the proposed EKNN model outperforms other diagnostic models because it introduces the best performance values.

**Table 5:** The performance measures values for diagnostic methods at training cases number = 100

Diagnostic methods	KNN	NB	SVM	DLA	EKNN
Accuracy (%)	62	70	72	79	86
Error (%)	38	30	28	21	14
Precision (%)	55	60	63	70	72
Recall (%)	57	62	65	67	76
Micro-average precision (%)	57	62	65	75	77
Macro-average precision (%)	61	62	62	70	73
Micro-average recall (%)	54	59	63	65	71
Macro-average recall (%)	59	59	60	65	67
F1-measure (%)	60	62	65	69	70
Execution time (Sec.)	6	5.5	5	4.5	2.5

Figures (9, 10, 11, 12, 13, 14, 15, 16, 17, 18) and Table 5 show that EKNN outperforms classical KNN, NB, SVM, and DLA as it can provide the best performance. It can achieve a maximum accuracy of 86%, a minimum error of 14%, and a minimum implementation time of 2.5 s when the number of training data = 100. On the other hand, the worst performance is provided by KNN where it provides a minimum accuracy of 62%, a maximum error of 38%, and a maximum implementation time of 6 s. Hence, the best performance is provided by EKNN while the worst performance is provided by KNN. According to Figs. (9, 10, 11, 12, 13, 14, 15, 16, 17, 18) and Table 5, the second and third best methods after EKNN are DLA and SVM respectively. Hence, the three methods of EDM called SVM, DLA, and EKNN are the best methods compared to classical KNN and NB methods. Based on EDM methods, it is noted that EKNN outperforms SVM and DLA. When the number of training data = 100, Fig. 9 and Table 5 show that the accuracy values of KNN, NB, SVM, DLA, and EKNN are 62%, 70%,72%,79%, and 86% respectively. Figure 10 and Table 5 illustrate that the error values of KNN, NB, SVM, DLA, and EKNN are 38%, 30%, 28%, 21%, and 14% respectively at the number of training data = 100.

When the number of training cases = 100 in Fig. 11 and Table 5, the precision values of KNN, NB, SVM, DLA, and EKNN are 55%, 60%, 63%, 70%, and 72% respectively. Figure 12 and Table 5 show that the recall values of KNN, NB, SVM, DLA, and EKNN at the number of training data = 100 are 57%, 62%, 65%, 67%, and 76% respectively. Thus, the best precision and recall values are given by EKNN but the worst value is given by KNN. In Fig. 13 and Table 5, the micro-average precision values of KNN, NB, SVM, DLA, and EKNN are 57%, 62%, 65%, 75%, and 77% respectively at training cases number = 100. Figure 14 and Table 5 show that the macro-average precision values of KNN, NB, SVM, DLA, and EKNN are 61%, 62%, 62%, 70%, and 73% respectively at the maximum number of training cases. According to the micro-average recall values in Fig. 15 and Table 5, KNN, NB, SVM, DLA, and EKNN reach to 54%, 59%, 63%, 65%, and 71% respectively at the maximum number of training cases. In Fig. 16 and Table 5, the macro-average recall values for the same techniques in the same order are 59%, 59%, 60%, 65%, and 67% respectively at the maximum number of training cases. Figure 17 and Table 5 show that the F1-measure of KNN, NB, SVM, DLA, and EKNN are 60%, 62%, 65%, 69%, and 70% respectively at training cases number = 100.

Figure 18 and Table 5 show that the implementation time of KNN, NB, SVM, DLA, and EKNN are 6, 5.5, 5, 4.5, and 2.5 s respectively at the training cases number = 100. It is noted in Fig. 18 that, EKNN is the fast method while KNN is the slow method. At the end, it is concluded that the performance of EKNN is superior to KNN, NB, SVM, and DLA. Thus, EKNN will be used in the DASD strategy to provide a fast and more accurate results.

### Testing diagnostic autism spectrum disorder (DASD) strategy

Through this subsection, DASD as a new diagnostic strategy will be tested and compared to other strategies called DL_ Xception [2], RF [5], ANN [6], DCNN [7], and LRM [1]. Three main steps are followed to implement the DASD strategy. In the first step, the BGWO method is executed to identify the useful set of features and then the BGA is executed in the second step to remove invalid training data. In the third and final step, EKNN is implemented on the filtered data to provide a quick and correct results. The accuracy, error, recall, precision, micro and macro average for precision, micro and macro average for recall, F1-measure, and implementation-time of these diagnostic strategies are showed in Figs. (9, 10, 11, 12, 13, 14, 15, 16, 17, 18) and Table 6. In fact, the proposed DASD strategy outperforms other strategies because it introduces the best performance values.

**Table 6:** The performance measures values for the used diagnostic strategies at training cases number = 100

Diagnostic Methods	DL_Xception	RF	ANN	DCNN	LRM	DASD
Accuracy (%)	82	85	87	89	90	93
Error (%)	18	15	13	12	10	7
Precision (%)	58	62	65	71	75	82
Recall (%)	60	62	64	72	75	83
Micro-average precision (%)	61	62	67	70	73	80
Macro-average precision (%)	65	64	62	70	73	83
Micro-average recall (%)	54	60	64	66	72	79
Macro-average recall (%)	56	62	62	69	75	81
F1-measure (%)	51	58	61	69	72	79
Execution time (Sec.)	9	6	5	4.5	4	1.5

**Fig. 19:**
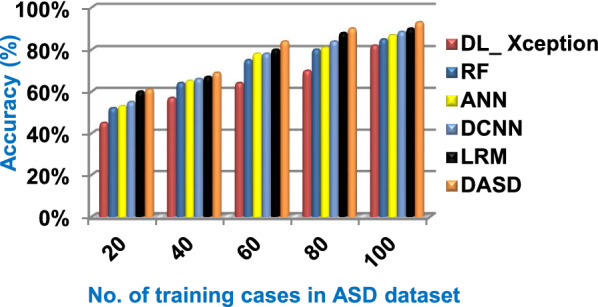
Accuracy of the used strategies

**Fig. 20:**
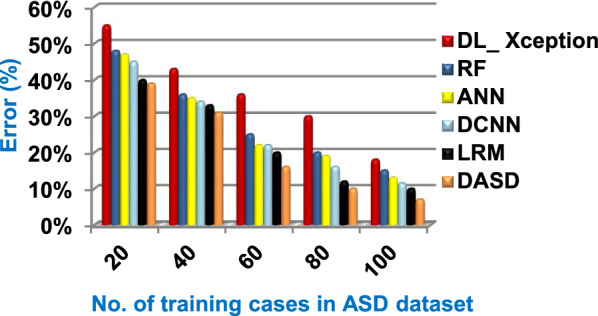
Error of the used strategies

**Fig. 21:**
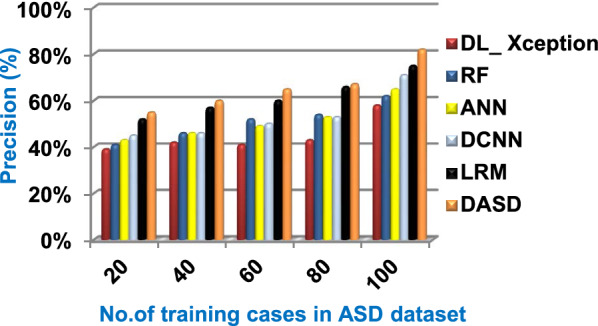
Precision of the used strategies

**Fig. 22:**
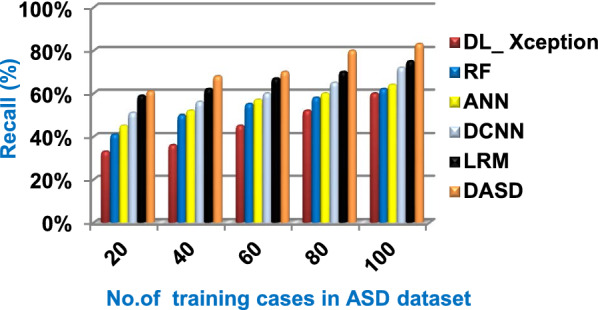
Recall of the used strategies

Figures (9, 10, 11, 12, 13, 14, 15, 16, 17, 18) and Table 6 illustrate that DASD strategy is superior than other diagnostic strategies, which are; DL_ Xception, RF, ANN, DCNN, and LRM. The reason is that DASD strategy provides a maximum accuracy with value equal 93%, a minimum error with value equal 7%, and a minimum implementation time with value equal 1.5 s when the training cases number = 100. Otherwise, DL_ Xception provides the worst performance because it introduces a minimum accuracy with value equal 82%, a maximum error with value equal 18%, and a maximum implementation time of 9 s. Figure 19 and Table 6 show that the accuracy values of DL_ Xception, RF, ANN, DCNN, LRM, and DASD are 82%, 85%,87%,88.5%, 90%, and 93% respectively at the training cases number = 100. On the other hand, the error values of DL_ Xception, RF, ANN, DCNN, LRM, and DASD in Fig. 20 and Table 6 are 18%, 15%, 13%, 11.5%, 10%, and 7% respectively at the training cases number = 100. According to Fig. 21 and Table 6, DL_ Xception, RF, ANN, DCNN, LRM, and DASD provide precision values reach to 58%, 62%, 65%, 71%, 75%, and 82% respectively at the training cases number = 100. DL_ Xception, RF, ANN, DCNN, LRM, and DASD provide recall values reach to 60%, 62%, 64%, 72%, 75%, and 83% at the number of training data = 100 as shown in Fig. 22 and Table 6. Hence, DL_ Xception gives the minimum precision and recall values and DASD provides the maximum values.

**Fig. 23:**
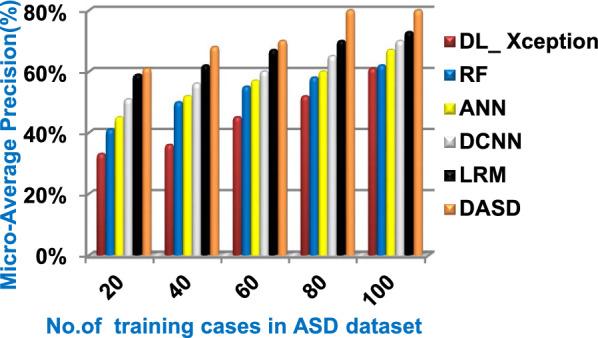
Micro-average precision of the used strategies

**Fig. 24:**
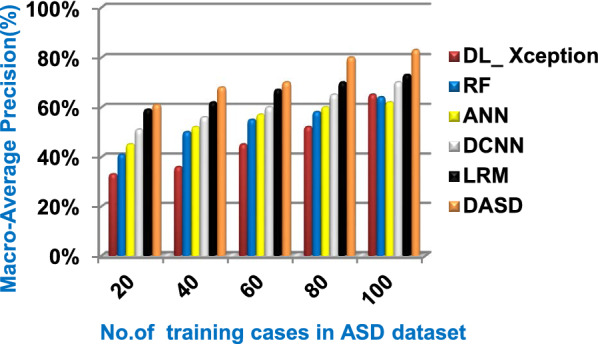
Macro-average precision of the used strategies

**Fig. 25:**
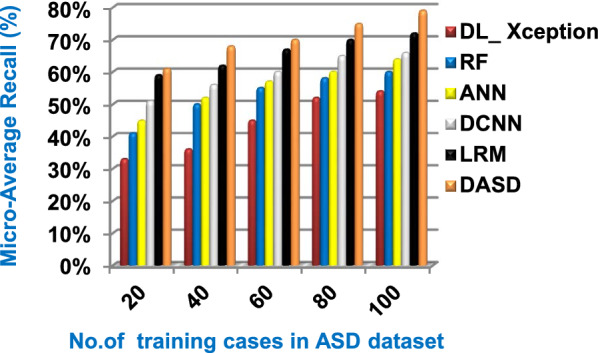
Micro-average recall of the used strategies

**Fig. 26:**
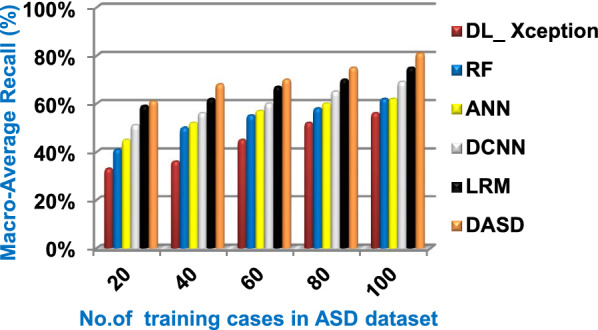
Macro-average recall of the used strategies

**Fig. 27:**
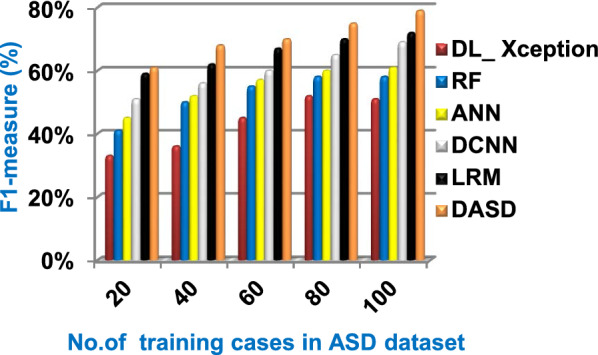
F1-measure of the used strategies

**Fig. 28:**
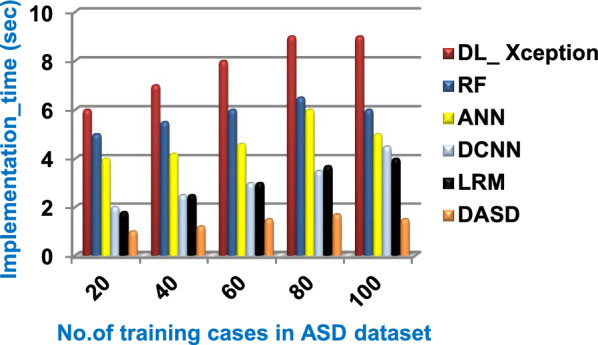
Implementation time of the used strategies

According to Fig. 23 and Table 6, it is noted that the micro-average precision of DL_ Xception, RF, ANN, DCNN, LRM, and DASD are 61%, 62%, 67%, 70%, 73%, and 80% respectively. Hence, the best micro-average precision value is provided by the proposed DASD while the worst value is provided by DL_ Xception. In Fig. 24 and Table 6, the macro-average precision of DL_ Xception, RF, ANN, DCNN, LRM, and DASD are 65%, 64%, 62%, 70%, 73%, and 83% respectively. Consequently, the maximum macro-average precision value is provided by DASD while the minimum value is provided by ANN. Figure 25 and Table 6 show that the micro-average recall of DL_ Xception, RF, ANN, DCNN, LRM, and DASD are 54%, 60%, 64%, 66%, 72%, and 79% respectively. The macro-average recall of these strategies in the same order are 56%, 62%, 62%, 69%, 75%, and 81% respectively as provided in Fig. 26 and Table 6. The best macro-average recall and micro-average recall values are given by DASD but the worst values are given by DL_ Xception. In Fig. 27 and Table 6, F1-measure of DL_ Xception, RF, ANN, DCNN, LRM, and DASD are 51%, 58%, 61%, 69%, 72%, and 79% respectively. Thus, DASD provides the best F1-measure value while DL_ Xception provides the worst value. The implementation time of these strategies is shown in Fig. 28 and Table 6. The implementation of DL_ Xception, RF, ANN, DCNN, LRM, and DASD takes time equal 9, 6, 5, 4.5, 4, and 1.5 s respectively at the training cases number = 100.

Consequently, the speed of DASD is faster than DL_ Xception, RF, ANN, DCNN, and LRM as DASD is based on applying pre-processing stage containing BGWO as a feature selection method and BGA as an outlier rejection method to filter data before starting to learn EKNN model. At the end, it is concluded that the performance of DASD method outperforms DL_ Xception, RF, ANN, DCNN, and LRM. That is because it provides 93%, 7%, 83%, 82%, 80%, 83%, 79%, 81%, 79%, and 1.5 s for accuracy, error, recall, precision, micro-average precision, macro-average precision, micro-average recall, macro-average recall, F1-measure, and implementation time respectively.

Based on the previous results, it can be concluded that the proposed DASD strategy outperformed all other strategies. This happened because DASD takes its decision based on the proposed EDM that combines the benefits of three different diagnostic methods, namely; SVM, DLA, and EKNN. Hence, it has the ability to give a final accurate diagnosis. In fact, EDM combines evidence from SVM as an associated learning method, DLA as a machine learning method, and EKNN as a distance based method. Combining these three different types of classifiers, which depend on different heuristics guarantee a high diagnosing efficiency as one classifier can compensate the deficiencies or shortcomings of the other classifiers. Finally, the diagnosing decision can be taken by combining the results of these three methods to provide fast and more accurate result. Hence, the presented DASD strategy, which is based on the proposed EDM, can be relied upon to provide accurate diagnostic decisions.

## The pros and cons of the proposed DASD strategy

According to experimental results, there are several pros and cons of DASD strategy. In fact, DASD can provide quick and accurate results. Also, DASD is a scalable strategy that has a high efficiency. The reason that the DASD strategy depends on a new proposed diagnostic method called EDM that combines the benefits of three different classifiers, namely; SVM, DLA, and the proposed EKNN after removing outliers and irrelevant features from the employed dataset. Although the benefits of DASD, it is a complex strategy that is only applied on binary label data and also applied on a small dataset. Table 7 summarizes the pros and cons of ASDD strategy.

**Table 7:** The pros and cons of DASD strategy

Pros		Cons	
Feature	Description	Feature	Description
Speed	DASD can quickly diagnose ASD cases because the diagnostic technique is used based on filtered data without invalid data	Size of Dataset	DASD was tested on small size of dataset
Accuracy	DASD can accurately diagnose ASD cases based on valid data	Complexity	DASD is a complex strategy as it contains prepressing and diagnosis phases.
Efficiency	DASD has a high effectiveness as it can give quick and accurate results	Multi-label classification	DASD is applied as a binary diagnostic strategy that can diagnose cases into ASD and TD
Number of features	DASD can handle large number of features		

## Conclusions and future directions

In this paper, Diagnostic Autism Spectrum Disorder (DASD) strategy has been provided to correctly detect ASD children. DASD composes of Data Filter Layer (DFL) and Diagnostic Layer (DL). In DFL, Binary Gray Wolf Optimization (BGWO) method was used to select the most significant features and Binary Genetic Algorithm (BGA) method was used to remove any outliers in the ASD dataset. Then, the filtered data was followed to DL to accurately learn the proposed Ensemble Diagnosis Methodology (EDM) to give fast and accurate diagnosis. In fact, EDM consists of three main diagnostic models called Support Vector Machine (SVM), Deep Learning Algorithm (DLA), and the proposed Enhanced K-Nearest Neighbors (EKNN) model. Related to experimental results, EKNN outperformed SVM and DLA models as it can provide accurate results in minimal execution time. Thus, EKNN was used in the DASD strategy depending on the current dataset to give a prompt and correct diagnosis. Accordingly, the DASD strategy provided satisfied results. This is because DASD provided maximum accuracy, recall, and precision while had minimum error and implementation time compared to other diagnostic strategies with values equal to 93%, 7%, 83%, 82%, 80%, 83%, 79%, 81%, 79%, and 1.5 s respectively, when trained on the maximum training cases number. Thus, it is noted that the DASD strategy provided the best accuracy value against other strategies.

There are many directions which will be taken in the future to improve the proposed DASD strategy. The DASD will be implemented on several datasets at different sizes. In DFL, the feature selection method should be improved by integrating it to a quick selection approach to enable the BGWO to provide a quick and accurate set of features. Additionally, the outlier rejection approach should be improved by integrating it to a quick rejection method to enable the BGA to quickly and accurately remove outliers. The most important applications of the proposed DASD strategy introduced in this paper can be summarized in the following points:

- Introducing diagnostic devices that can diagnose many different diseases based on AI such as diagnosing different types of cancer, COVID-19, monkeypox, heart diseases, liver diseases,….. etc..
- Developing intelligent robots based on AI that can set with ASD cases to help them in their early recovery because the delay in diagnosing ASD cases will make it difficult to deal with or treat them.
- Developing diseases treatment plans.
